# Supplementary material for: Polyoxometalate-based plasmonic electron sponge membrane for nanofluidic osmotic energy conversion
Source: Nat Commun. 2024 May 17;15:4213. doi: 10.1038/s41467-024-48613-6 (PMC11101624; doi:10.1038/s41467-024-48613-6)
Supplement: Supplementary file 1 — Supplementary Information [file 41467_2024_48613_MOESM1_ESM.pdf]

Supplementary Information for Polyoxometalate-Based Plasmonic Electron Sponge Membrane for Nanofluidic Osmotic Energy Conversion

Chengcheng Zhu,[1] Li Xu,[1] Yazi Liu,[2] Jiang Liu,[3] Jin Wang,[1,4] Hanjun Sun,[1] Ya-Qian Lan,[1,3]* Chen Wang[1]*

[1]Jiangsu Key Laboratory of New Power Batteries, School of Chemistry and Materials Science, Nanjing Normal University, Nanjing 210023, China

[2]School of Environment, Jiangsu Engineering Lab of Water and Soil Eco-remediation, Nanjing Normal University, Jiangsu Engineering Lab of Water and Soil Eco-remediation, Nanjing Normal University, Nanjing Normal University, Nanjing 210023, China.

[3]School of Chemistry, South China Normal University, Guangzhou 510006, China

*To whom correspondence should be addressed.

E-mail: yqlan@m.scnu.edu.cn; wangchen@njnu.edu.cn

Table of Contents

Supporting Figures and Tables
Supplementary Figure 1. (A&B) TEM images of 35nm AuNPs.
Supplementary Figure 2. The W 4*f* XPS spectra of PW_{12} and Au@PW_{12}.
Supplementary Figure 3. TEM images of Au@POMs.
Supplementary Figure 4. Zeta potential of Au and Au@POMs.
Supplementary Figure 5. SEM images of the top of pure AAO membrane.
Supplementary Figure 6. The SEM image of the cross-section of pure AAO membrane.
Supplementary Figure 7. Large-area uniform monolayer Au@POMs nanofilm transferred once on the Silicon wafer.
Supplementary Figure 8. SEM images of the top (A1-A4) and cross-section (B1-B4) of PESM with different thickness (the Au@POMs layer was transferred for once, twice, three times, and four times respectively.
Supplementary Figure 9. I-V curves of PESM with different thickness.
Supplementary Figure 10. ICR ratio of PESM with different thickness.
Supplementary Figure 11. Current density of PESM with different thickness. The inset is power density of PESM with different thickness.
Supplementary Figure 12. Power density of PESM with different thickness under light and no light irradiation.
Supplementary Figure 13. The photograph of the PESM membrane.
Supplementary Figure 14. (A-D) The SEM images of the PESM membrane.
Supplementary Figure 15. I-V curves of PESM in 1M KCl solution (pH=7).
Supplementary Figure 16. Current of AAO, Au/AAO and PESM.
Supplementary Figure 17. ICR ratio of AAO, Au/AAO and PESM.
Supplementary Figure 18. I-V curves of PESM measured in a 100 mM KCl solution at different pH values.
Supplementary Figure 19. I-V curves of the PESM in various concentrations of KCl solution.
Supplementary Figure 20. I-V curves of the PESM in various concentrations of K_2SO_4 solution.
Supplementary Figure 21. I-V curves of the PESM in various concentrations of $BaCl_2$ solution.
Supplementary Figure 22. (A) I-V curves and (B) ICR ratio of the PESM in various concentrations of NaCl solution.
Supplementary Figure 23. The (A)I-V curves and (B) ICR ratio of PESN in various concentrations of $MgCl_2$ solution.
Supplementary Figure 24. Current changes at different voltage values under light irradiation.
Supplementary Figure 25. Contact angle of PESM with and without light irradiation, respectively.
Supplementary Figure 26. Power density AAO, Au/AAO and PESM.
Supplementary Figure 27. (A) Current density and (B) power density of PESM under a series of NaCl concentration gradient. (C) Current density and (D) power density of PESM under a series of KCl concentration gradient.

Supplementary Figure 28. (A) TEM image of 15 nm AuNPs. (B) TEM image of 50 nm AuNPs. (C) TEM image of Au@POMs using 15 nm AuNPs. (D) TEM image of Au@POMs using 50 nm AuNPs. (E) The HAADF-STEM image of Au@POMs using 15 nm AuNPs and corresponding energy-dispersive x-ray (EDX) elemental mappings of Au, P and W. (F) The HAADF-STEM image of Au@POMs using 50 nm AuNPs and corresponding energy-dispersive x-ray (EDX) elemental mappings of Au, P and W. (G) SEM image and EDX elemental mappings of the top of PESM based on 15 nm AuNPs. (H) SEM image and EDX elemental mappings of the top of PESM based on 50 nm AuNPs. (I) I-V curves of PESM based on 15 nm, 35 nm and 50 nm AuNPs. (J) ICR ratio of PESM based on 15 nm, 35 nm and 50 nm AuNPs. (K) The current and power density of PESM based on 15 nm, 35 nm and 50 nm AuNPs (10 mM/500 mM NaCl).
Supplementary Figure 29. Conductance of PESM under different temperature.
Supplementary Figure 30. Short-circuit current of PESM under different temperature.
Supplementary Figure 31. (A) Current density and (B) power density of PESM under different temperature. Concentration gradient is 10 mM/500 mM NaCl.
Supplementary Figure 32. Open-circuit voltage of PESM under different temperature.
Supplementary Figure 33. Conductance of PESM under different pH.
Supplementary Figure 34. Open-circuit voltage of t PESM under different pH.
Supplementary Figure 35. Short-circuit current of PESM under different pH.
Supplementary Figure 36. (A) Current density and (B) power density of PESM under different pH. Concentration gradient is 10 mM/500 mM NaCl.
Supplementary Figure 37. The (A) current density and (B) power density of PESN under different light intensity.
Supplementary Figure 38. Power density of PESN under different light intensity.
Supplementary Figure 39. Current changes of AAO, Au/AAO and PESM with and without light irradiation, respectively.
Supplementary Figure 40. (A) Current density and (B) power density of AAO, Au/AAO and PESM with and without light irradiation, respectively. Concentration gradient is 10 mM/500 mM NaCl.
Supplementary Figure 41. Power density of AAO, Au/AAO and PESM with and without light irradiation, respectively. Concentration gradient is 10 mM/500 mM NaCl.
Supplementary Figure 42. IR camera images (532 nm, ~200 mW cm^{-2}) of Au/AAO.
Supplementary Figure 43. IR camera images (532 nm, ~200 mW cm^{-2}) of PESM.
Supplementary Figure 44. Power density of PESM with and without light irradiation under a series of KCl concentration gradients, respectively.
Supplementary Figure 45. (A) Current density and (B) power density of PESM with light irradiation under a series of KCl gradients.
Supplementary Figure 46. Power density of PESM under a series of NaCl concentration gradients with and without light irradiation.
Supplementary Figure 47. (A) Current density and (B) power density of PESM with light irradiation under different temperature. Concentration gradient is 10 mM/500 mM NaCl.
Supplementary Figure 48. Power density of PESM under different temperatures with and without light irradiation. Concentration gradient is 10 mM/500 mM NaCl.

Supplementary Figure 49. (A) Current density and (B) power density of PESM with light irradiation under different pH. Concentration gradient is 10 mM/500 mM NaCl.
Supplementary Figure 50. Power density of PESM under different pH with and without light irradiation. Concentration gradient is 10 mM/500 mM NaCl.
Supplementary Figure 51. Stability of PESM for ICR ratio.
Supplementary Figure 52. Stability of PESM under irradiation for energy conversion (50-fold NaCl).
Supplementary Figure 53. The photograph of the PESM membrane after 30-day usage.
Supplementary Figure 54. (A-D) The SEM images of the PESM membrane after 30-day usage.
Supplementary Figure 55. (A) The current density and (B) power density of the PESM membrane under natural seawater and river water.
Supplementary Figure 56. Cyclic voltammogram of the POMs using an Ag/AgCl reference electrode.
Supplementary Figure 57. UV-Vis diffuse-reflectance spectrum of POMs.
Supplementary Figure 58. Band gap of POMs estimated from UV-vis reflectance spectrum.
Supplementary Figure 59. Mott-Schottky plots of Au@POMs with and without light irradiation, respectively.
Supplementary Figure 60. UV-vis absorption spectra of AuNPs and Au@POMs (PW_{12}, P_2W_{18} and P_5W_{30}).
Supplementary Figure 61. TEM images of AuNPs and Au@POMs (PW_{12}, P_2W_{18} and P_5W_{30}).
Supplementary Figure 62. The W 4*f* XPS spectra of P_2W_{18} and Au@P_2W_{18}.
Supplementary Figure 63. The W 4*f* XPS spectra of P_5W_{30} and Au@P_5W_{30}.
Supplementary Figure 64. ICR ratio of PESM based on Keggin-type PW_{12}, Wells-Dawson-type P_2W_{18} and Preyssler-type P_5W_{30} with and without light irradiation, respectively.
Supplementary Figure 65. Current of PESM based on Keggin-type PW_{12}, Wells-Dawson-type P_2W_{18} and Preyssler-type P_5W_{30} with and without light irradiation, respectively.
Supplementary Figure 66. The cyclic voltammogram of the POMs (P_2W_{18}) using an Ag/AgCl reference electrode.
Supplementary Figure 67. The band gap of POMs (P_2W_{18}).
Supplementary Figure 68. The cyclic voltammogram of the POMs (P_5W_{30}) using an Ag/AgCl reference electrode.
Supplementary Figure 69. The band gap of POMs (P_5W_{30}).
Supplementary Figure 70. Schematic and energy level diagram illuminating hot-electron injection from Au NPs to POMs (including PW_{12}, P_2W_{18} and P_5W_{30}).
Supplementary Figure 71. (A) Current density and (B) power density of PESM based on Keggin-type PW_{12}, Wells-Dawson-type P_2W_{18} and Preyssler-type P_5W_{30} with and without light irradiation, respectively. Concentration gradient is 10mM/500mM NaCl.
Supplementary Figure 72. Power density of PESM based on Keggin-type PW_{12}, Wells-Dawson-type P_2W_{18} and Preyssler-type P_5W_{30} with and without light irradiation, respectively. Concentration gradient is 10mM/500mM NaCl.
Supplementary Figure 73. (A&B) TEM images of Au@PMo_{12}. (C) The HAADF-STEM image of Au@PMo_{12} and corresponding energy-dispersive x-ray (EDX) elemental mappings of Au, P and Mo. (D) SEM image of the top of PESM based on PMo_{12}. (E) SEM image of the cross-

section of PESM based on PMo_{12}. (F) I-V curves of PESM based on PMo_{12} in 1M KCl solutions with and without light irradiation. (G) The current and power density of PESM based on PMo_{12} with and without light irradiation (10 mM/500 mM NaCl).

Supplementary Figure 74. Colony growth after the static bacterial adhesion test (Gram-negative bacteria-E. coli).

Supplementary Figure 75. Colony growth after the static bacterial adhesion test (Gram-positive bacteria-S. aureus).

Supplementary Figure 76. Colony growth after the photoinduced germicidal test (Gram-positive bacteria-S. aureus).

Supplementary Figure 77. Diagram demonstrated the contributions of the different parts to the overall measure Isc and Vos. Salinity-gradient-driven power harvesting system based on PESM.

Supplementary Table 1. Corresponding I_{SC}, V_{OS}, E_{redox}, and E_{diff} under different concentration gradients.

Supplementary Table 2. Cation transfer number of PESM.

Supplementary Table 3. Comparison of output power density of PESM with reported different materials-based energy conversion systems.

Supplementary Table 4. TEM transmission electron microscopy (TEM) combined with energy-dispersive X-ray spectroscopy (EDS) analysis of Au@POMs using PW_{12}, P_2W_{18} and P_5W_{30} as ligands, respectively.

Supporting Figures and Tables

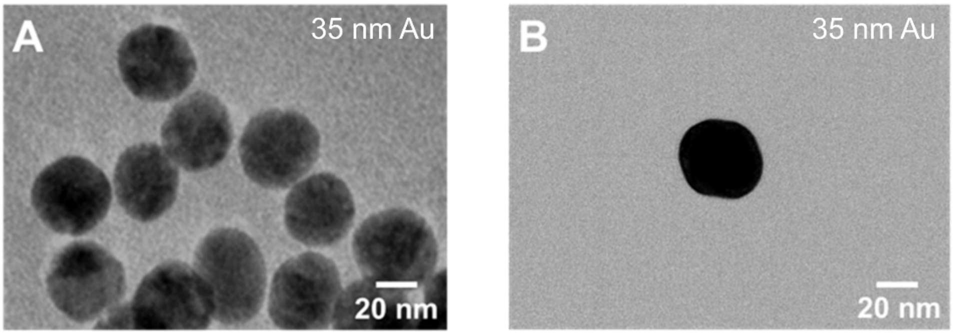

Supplementary Figure 1. (A&B) TEM images of 35nm AuNPs.

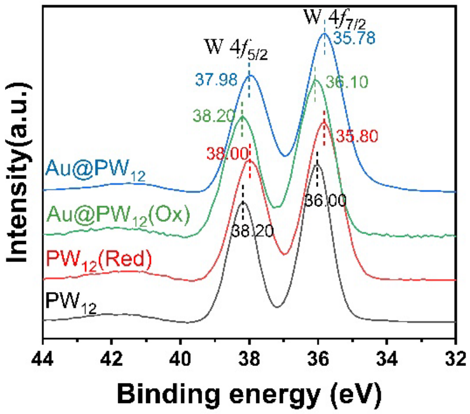

Supplementary Figure 2. The W 4*f* XPS spectra of PW_{12} and Au@PW_{12}.

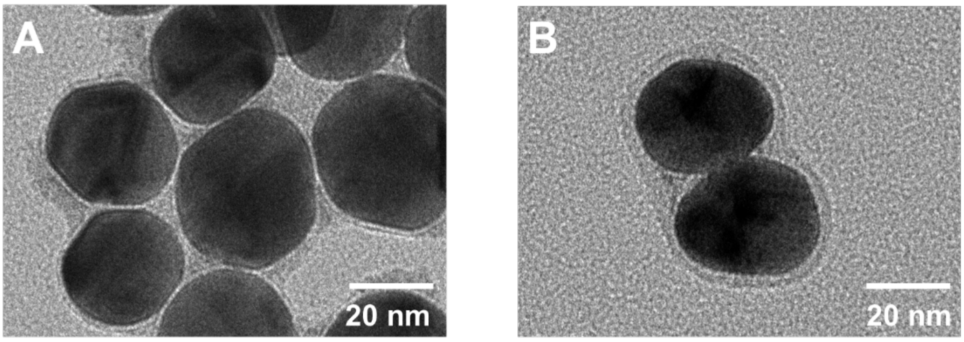

Supplementary Figure 3. TEM images of Au@POMs.

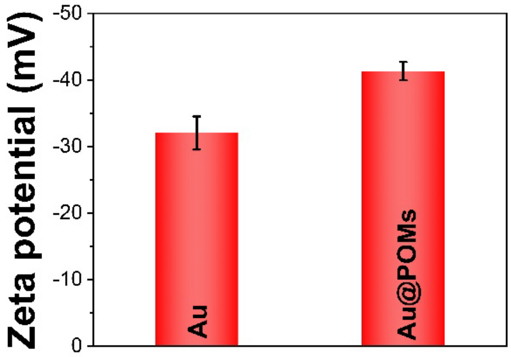

Supplementary Figure 4. Zeta potential of Au and Au@POMs. Error bars represent standard deviation of three different measurements.

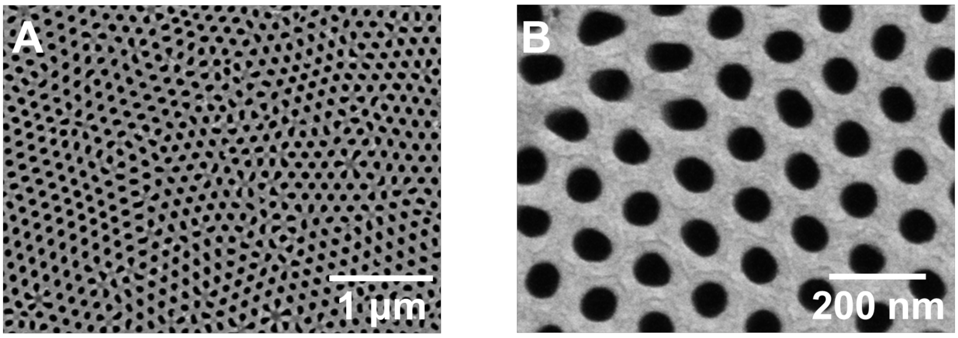

Supplementary Figure 5. SEM images of the top of pure AAO membrane.

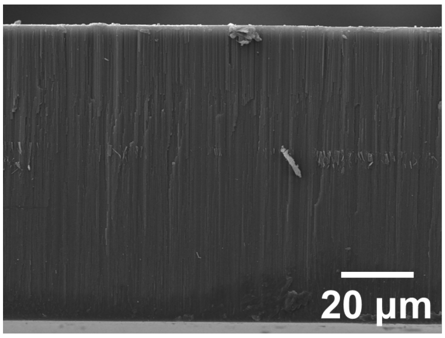

Supplementary Figure 6. The SEM image of the cross-section of pure AAO membrane.

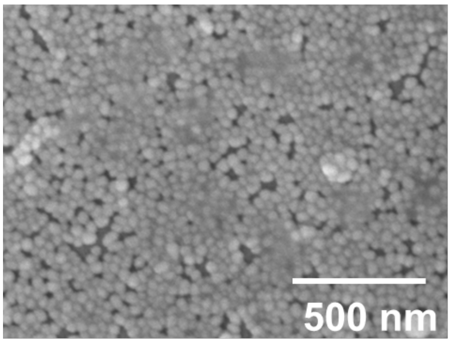

Supplementary Figure 7. Large-area uniform monolayer Au@POMs nanofilm transferred once on the Silicon wafer.

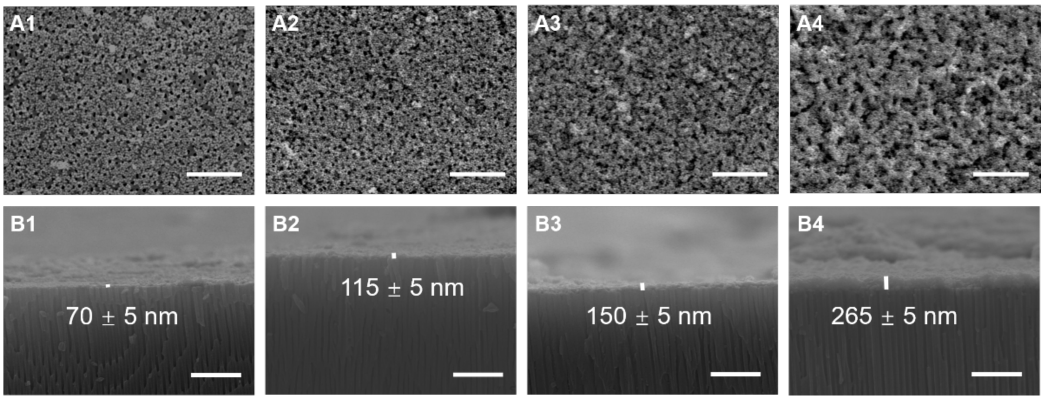

Supplementary Figure 8. SEM images of the top (A1-A4) and cross-section (B1-B4) of PESM with different thickness (the Au@POMs layer was transferred for once, twice, three times, and four times respectively. (scale bar is 1 μm)

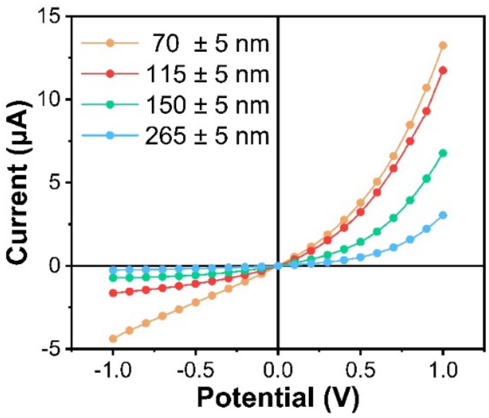

Supplementary Figure 9. I-V curves of PESM with different thickness.

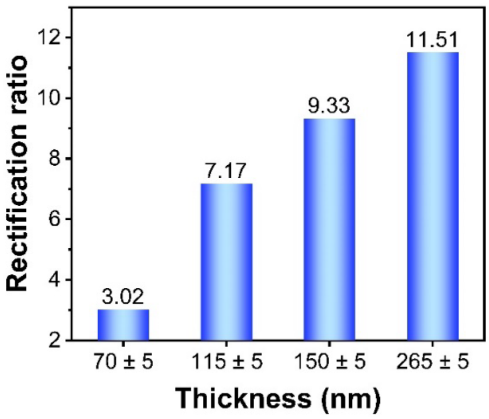

Supplementary Figure 10. ICR ratio of PESM with different thickness.

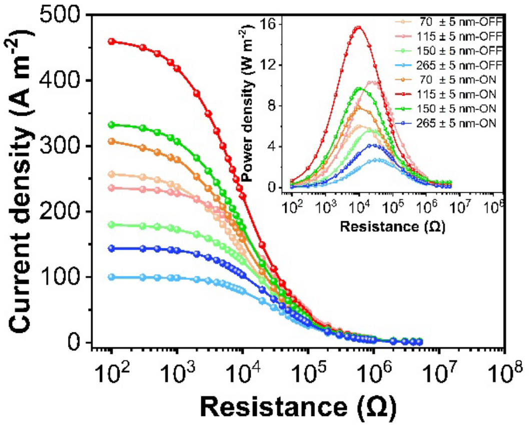

Supplementary Figure 11. Current density of PESM with different thickness. The inset is power density of PESM with different thickness.

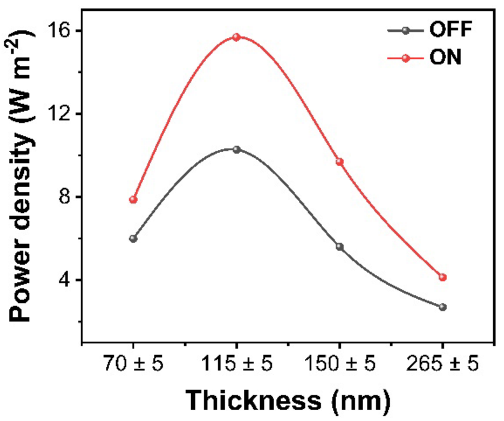

Supplementary Figure 12. Power density of PESM with different thickness under light and no light irradiation.

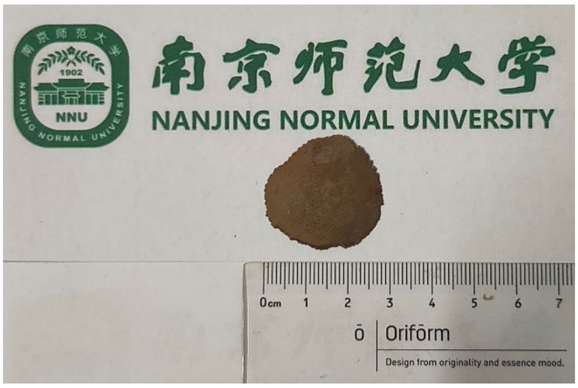

Supplementary Figure 13. The photograph of the PESM membrane.

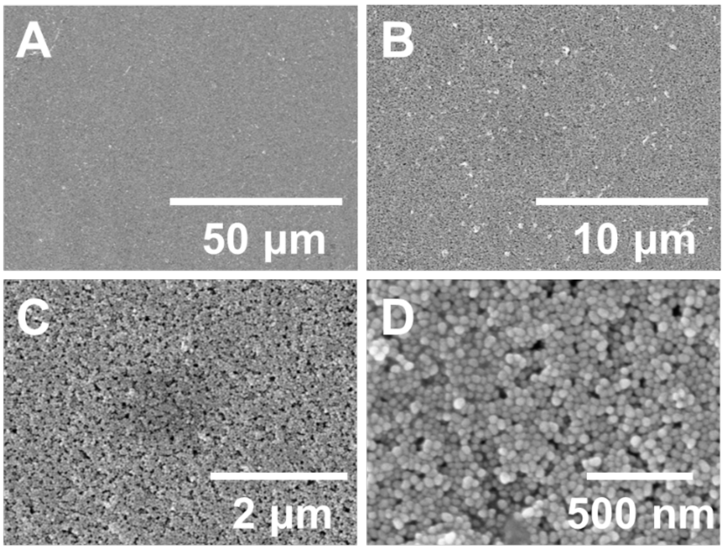

Supplementary Figure 14. S14. (A-D) The SEM images of the PESM membrane.

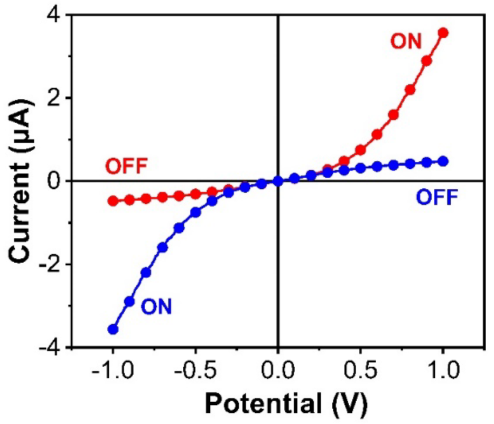

Supplementary Figure 15. I-V curves of PESM in 1 M KCl solution (pH=7).

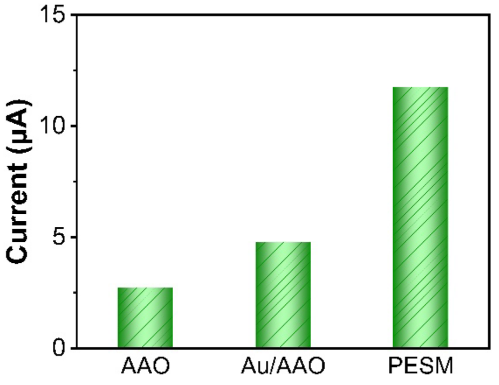

Supplementary Figure 16. Current of AAO, Au/AAO and PESM.

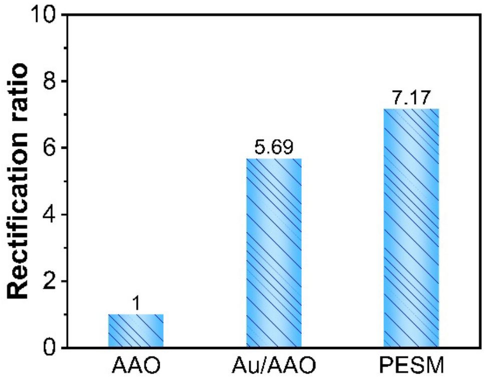

Supplementary Figure 17. ICR ratio of AAO, Au/AAO and PESM.

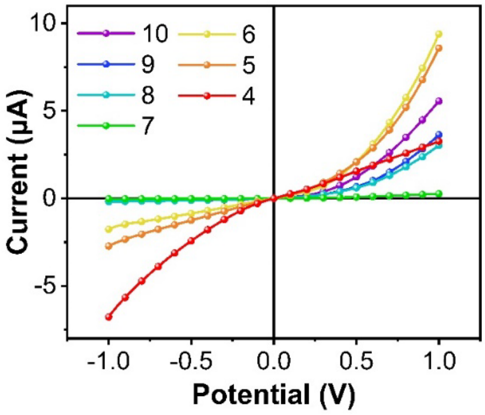

Supplementary Figure 18. I-V curves of PESM measured in a 100 mM KCl solution at different pH values.

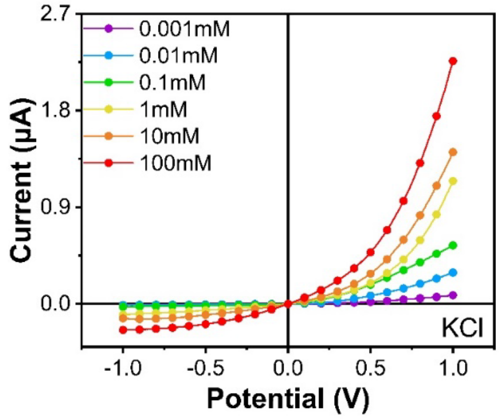

Supplementary Figure 19. I-V curves of the PESM in various concentrations of KCl solution.

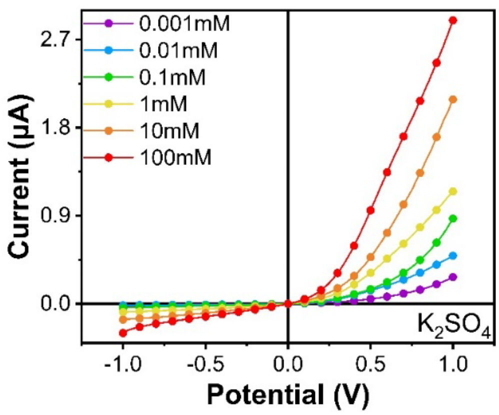

Supplementary Figure 20. I-V curves of the PESM in various concentrations of K_2SO_4 solution.

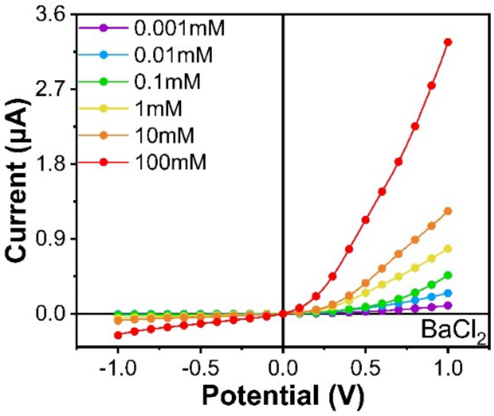

Supplementary Figure 21. I-V curves of the PESM in various concentrations of $BaCl_2$ solution.

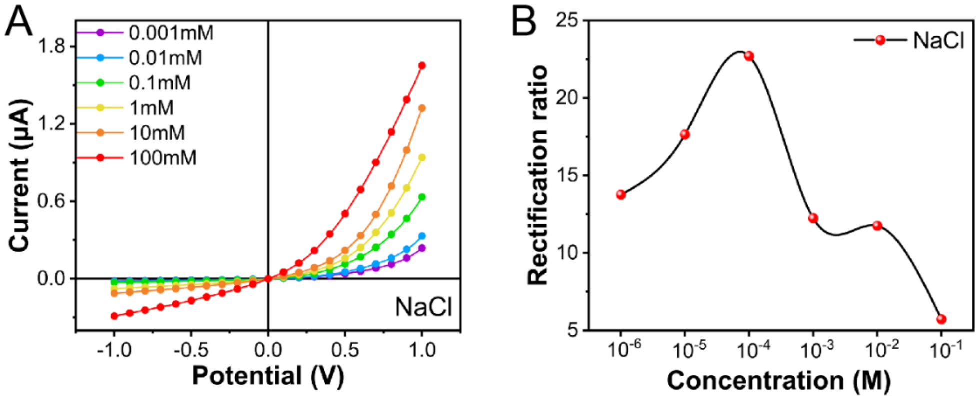

Supplementary Figure 22. (A) I-V curves and (B) ICR ratio of the PESM in various concentrations of NaCl solution.

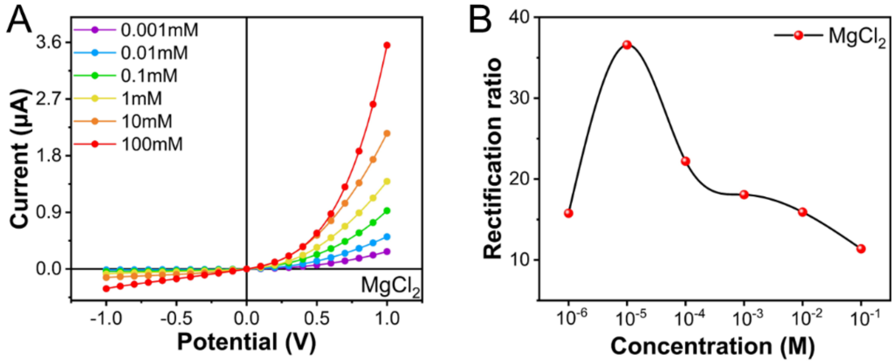

Supplementary Figure 23. The (A)I-V curves and (B) ICR ratio of PESN in various concentrations of $MgCl_2$ solution.

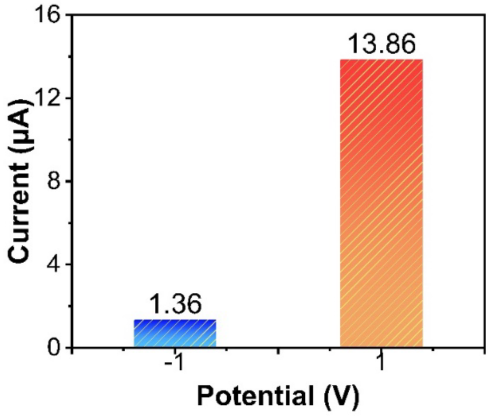

Supplementary Figure 24. Current changes at different voltage values under light irradiation.

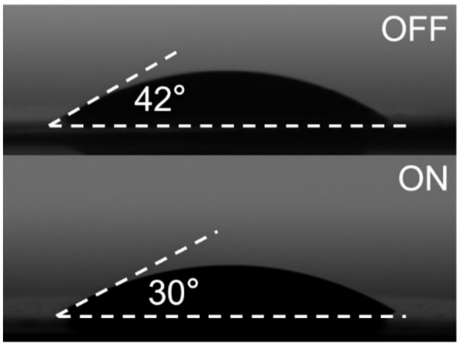

Supplementary Figure 25. Contact angle of PESM with and without light irradiation, respectively.

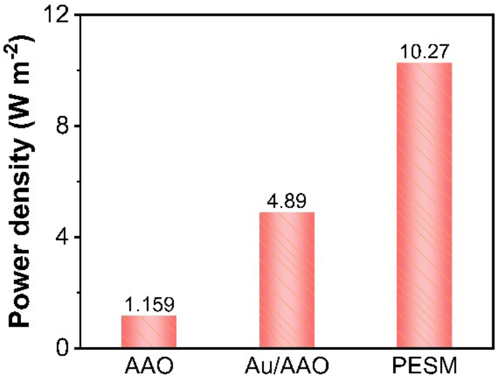

Supplementary Figure 26. Power density of AAO, Au/AAO and PESM.

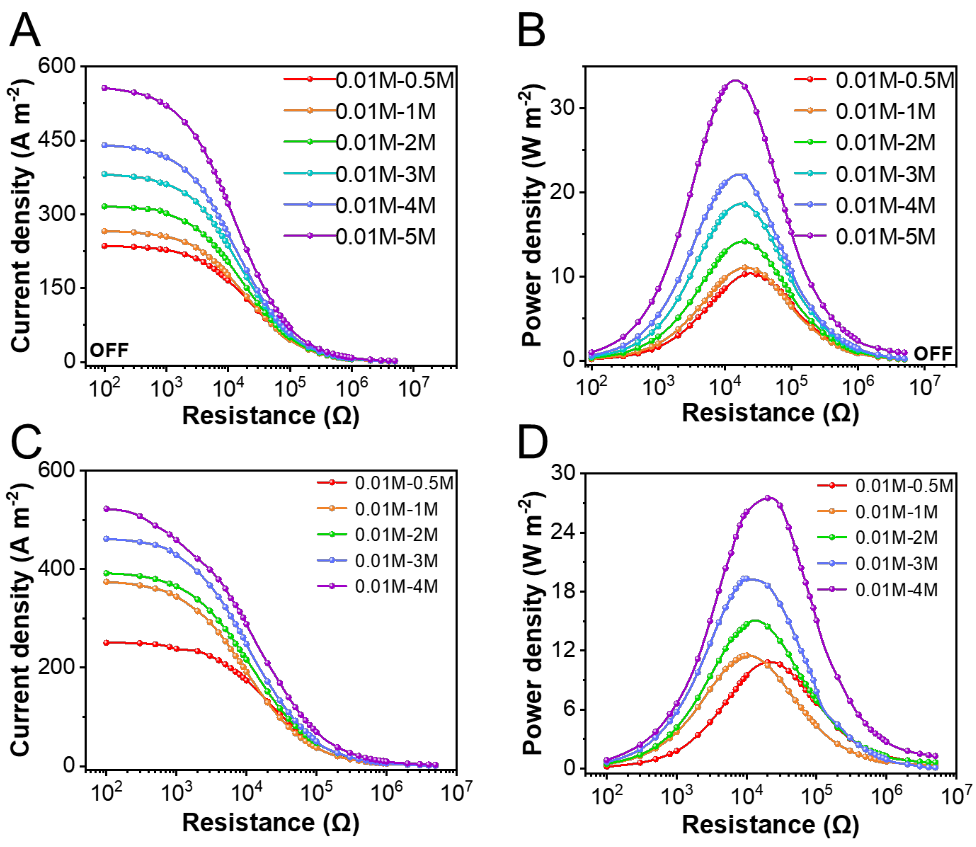

Supplementary Figure 27. (A) Current density and (B) power density of PESM under a series of NaCl concentration gradient. (C) Current density and (D) power density of PESM under a series of KCl concentration gradient.

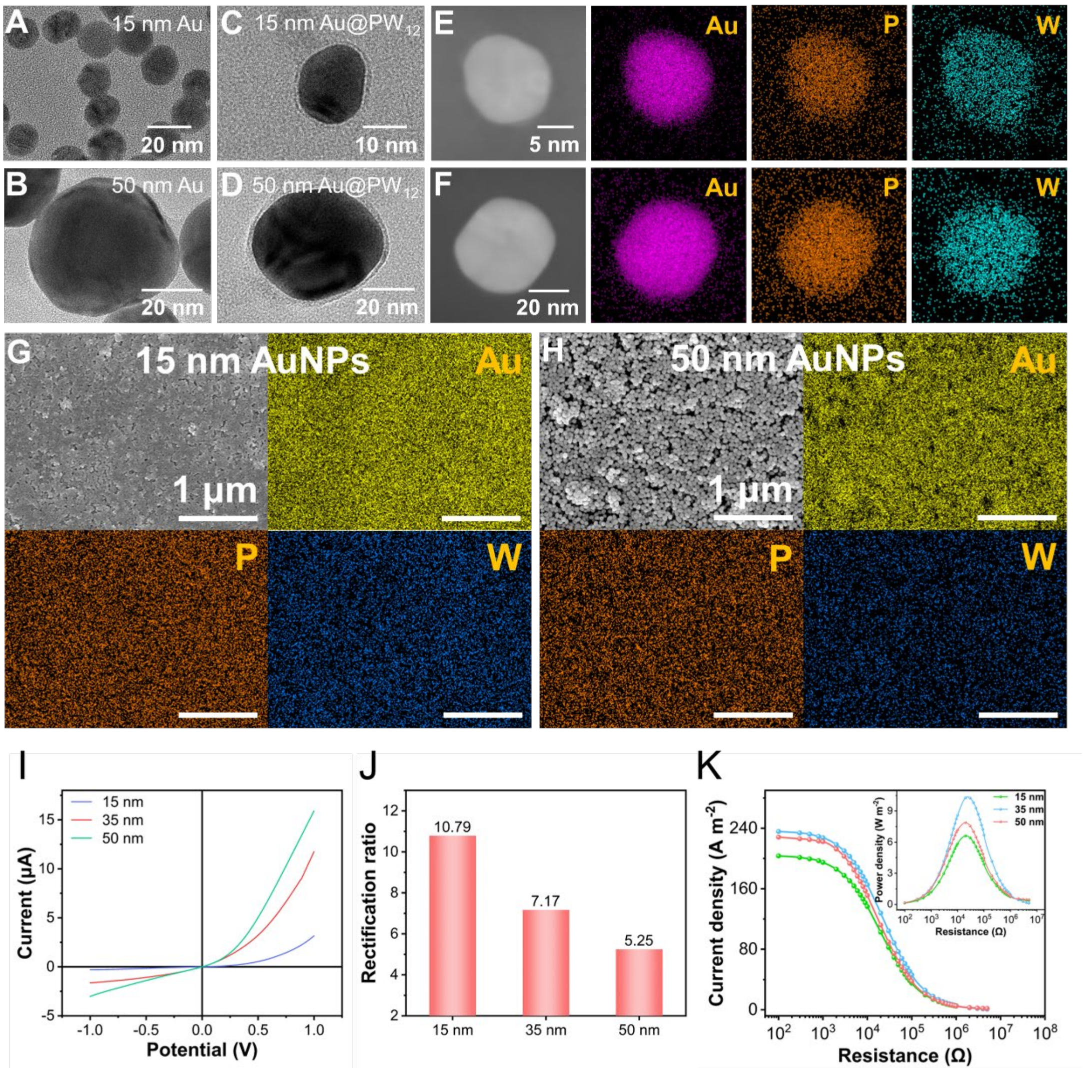

Supplementary Figure 28. (A) TEM image of 15 nm AuNPs. (B) TEM image of 50 nm AuNPs. (C) TEM image of Au@POMs using 15 nm AuNPs. (D) TEM image of Au@POMs using 50 nm AuNPs. (E) The HAADF-STEM image of Au@POMs using 15 nm AuNPs and corresponding energy-dispersive x-ray (EDX) elemental mappings of Au, P and W. (F) The HAADF-STEM image of Au@POMs using 50 nm AuNPs and corresponding energy-dispersive x-ray (EDX) elemental mappings of Au, P and W. (G) SEM image and EDX elemental mappings of the top of PESM based on 15 nm AuNPs. (H) SEM image and EDX elemental mappings of the top of PESM based on 50 nm AuNPs. (I) I-V curves of PESM based on 15 nm, 35 nm and 50 nm AuNPs. (J) ICR ratio of PESM based on 15 nm, 35 nm and 50 nm AuNPs. (K) The current and power density of PESM based on 15 nm, 35 nm and 50 nm AuNPs (10 mM/500 mM NaCl).

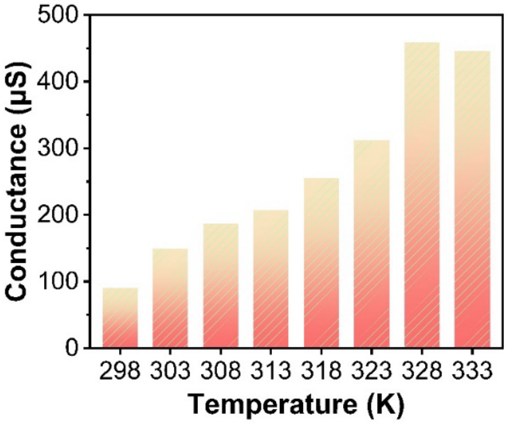

Supplementary Figure 29. Conductance of PESM under different temperature.

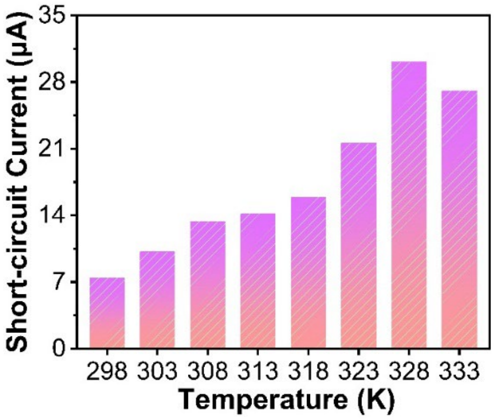

Supplementary Figure 30. Short-circuit current of PESM under different temperature.

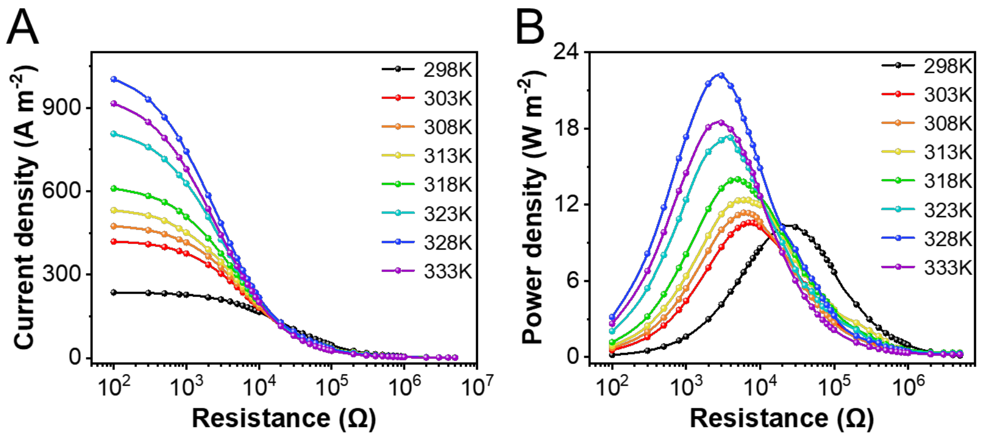

Supplementary Figure 31. (A) Current density and (B) power density of PESM under different temperature. Concentration gradient is 10 mM/500 mM NaCl.

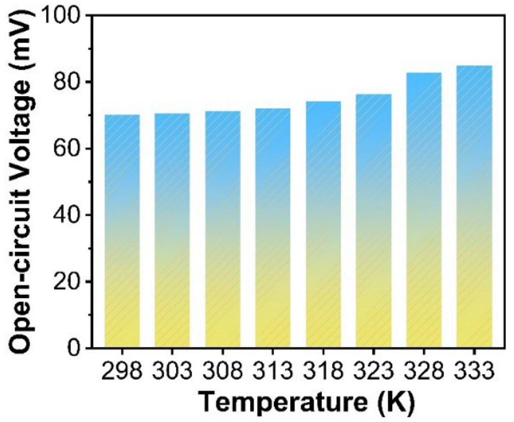

Supplementary Figure 32. Open-circuit voltage of PESM under different temperature.

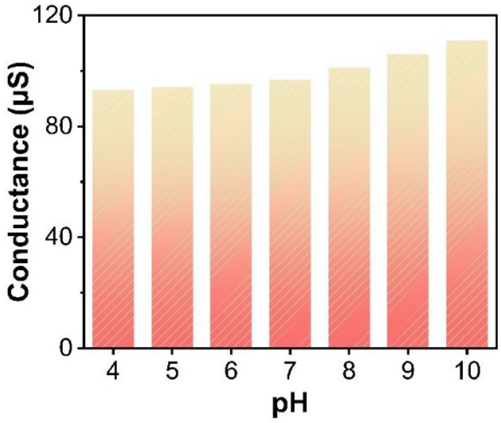

Supplementary Figure 33. Conductance of PESM under different pH.

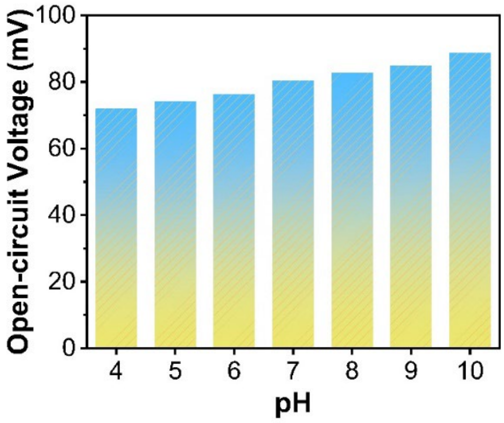

Supplementary Figure 34. Open-circuit voltage of t PESM under different pH.

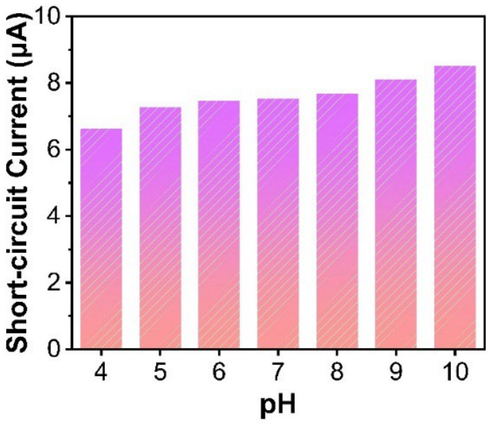

Supplementary Figure 35. Short-circuit current of PESM under different pH.

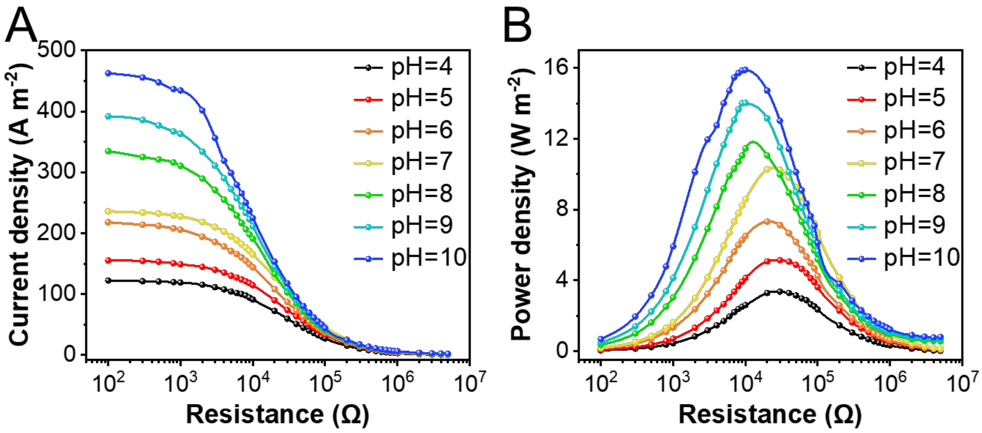

Supplementary Figure 36. (A) Current density and (B) power density of PESM under different pH. Concentration gradient is 10 mM/500 mM NaCl.

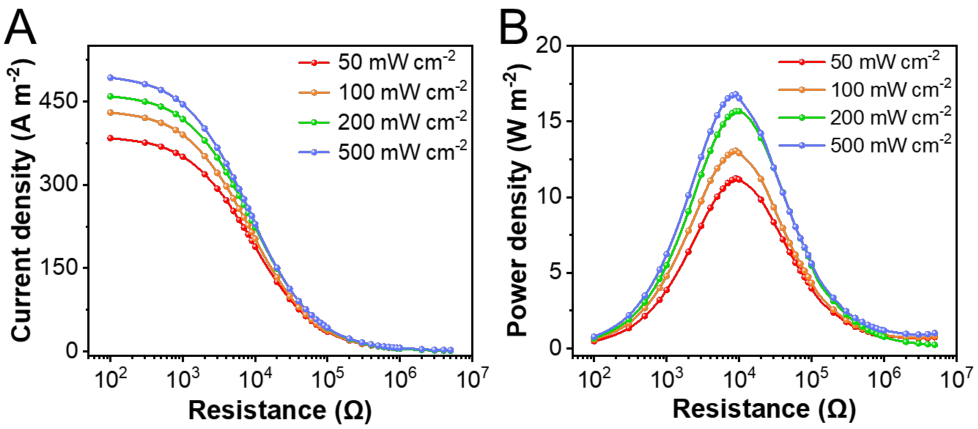

Supplementary Figure 37. The (A) current density and (B) power density of PESN under different light intensity.

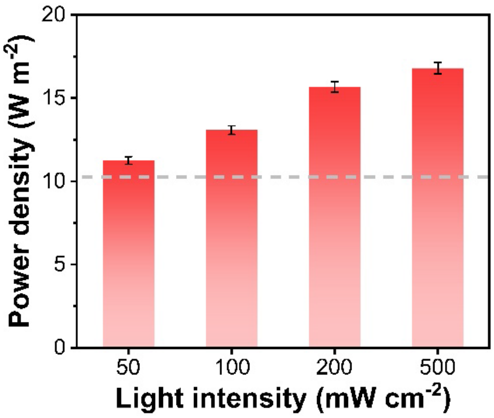

Supplementary Figure 38. Power density of PESN under different light intensity. Error bars represent standard deviation of three different measurements.

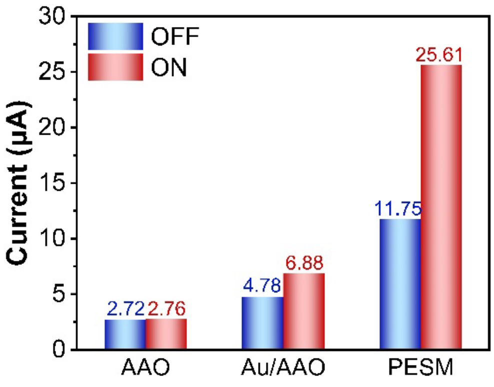

Supplementary Figure 39. Current changes of AAO, Au/AAO and PESM with and without light irradiation, respectively.

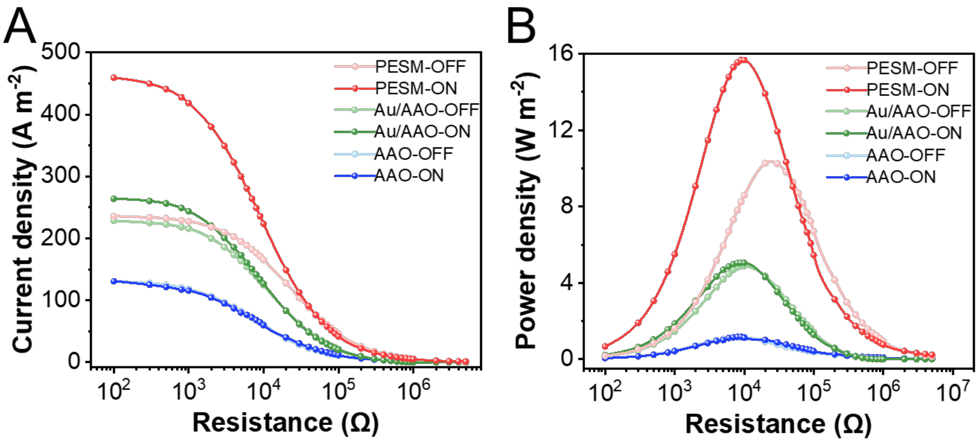

Supplementary Figure 40. (A) Current density and (B) power density of AAO, Au/AAO and PESM with and without light irradiation, respectively. Concentration gradient is 10 mM/500 mM NaCl.

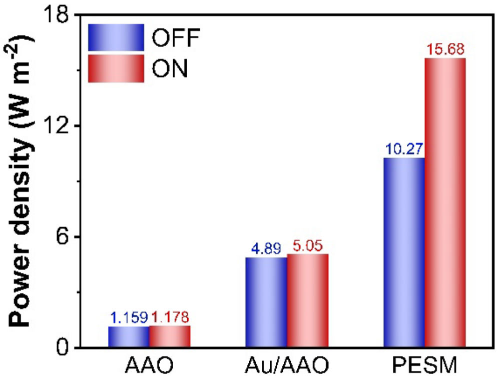

Supplementary Figure 41. Power density of AAO, Au/AAO and PESM with and without light irradiation, respectively. Concentration gradient is 10 mM/500 mM NaCl.

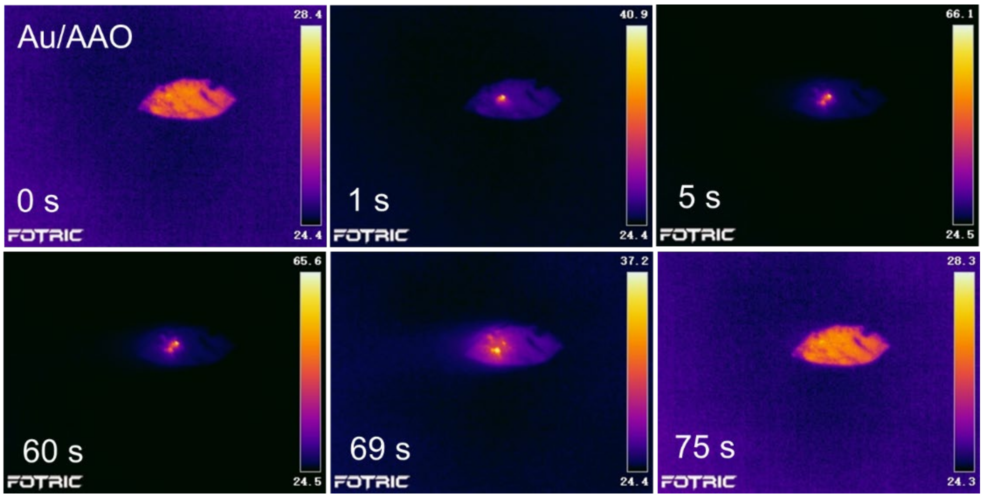

Supplementary Figure 42. IR camera images (532 nm, ~200 mW cm^{-2}) of Au/AAO.

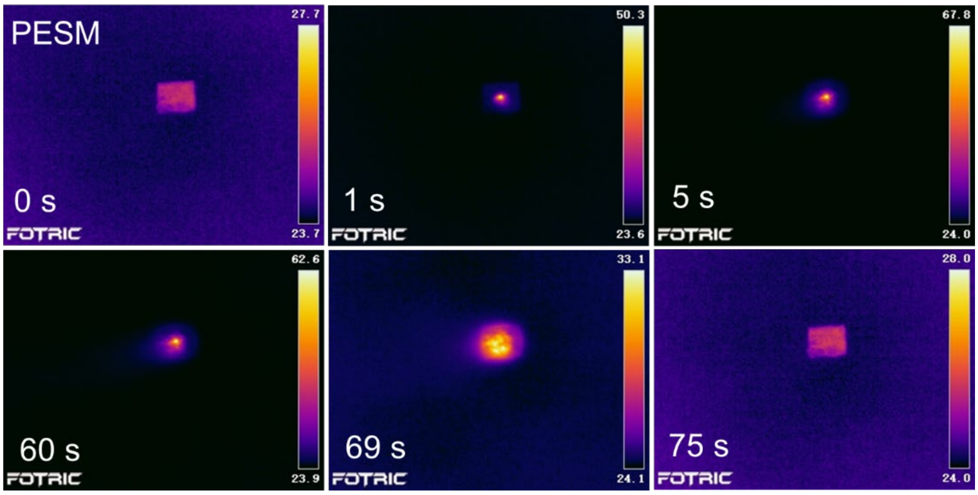

Supplementary Figure 43. IR camera images (532 nm, ~200 mW cm^{-2}) of PESM.

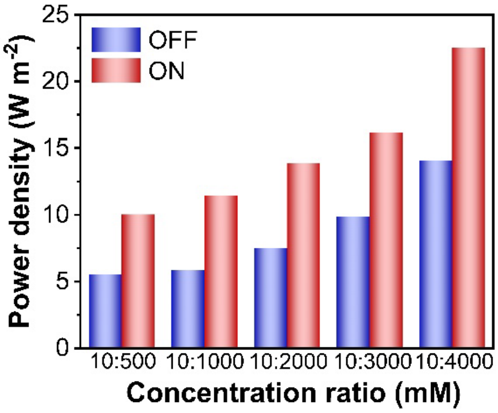

Supplementary Figure 44. Power density of PESM with and without light irradiation under a series of KCl concentration gradients, respectively.

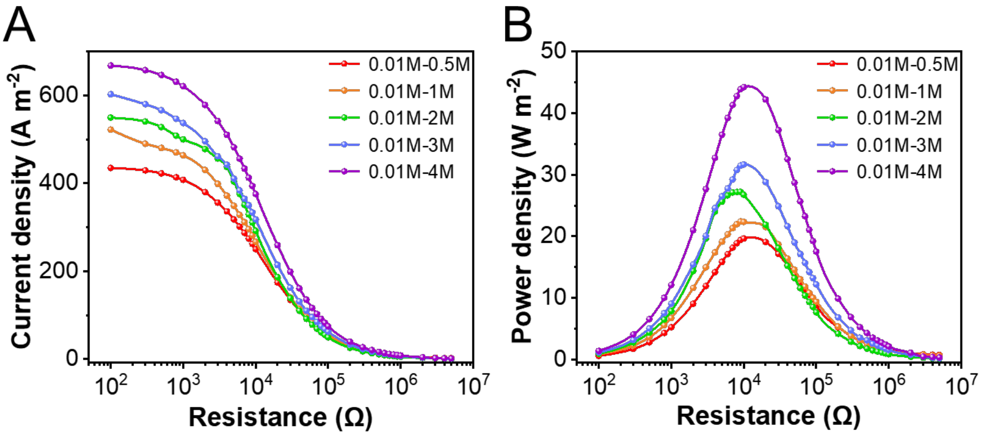

Supplementary Figure 45. (A) Current density and (B) power density of PESM with light irradiation under a series of KCl gradients.

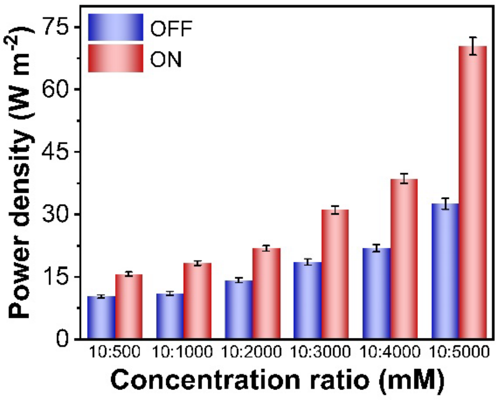

Supplementary Figure 46. Power density of PESM under a series of NaCl concentration gradients with and without light irradiation. Error bars represent standard deviation of three different measurements.

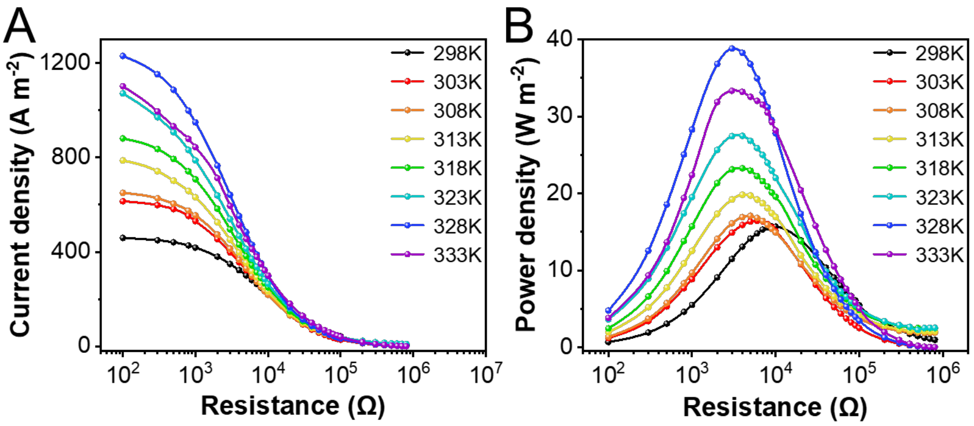

Supplementary Figure 47. (A) Current density and (B) power density of PESM with light irradiation under different temperature. Concentration gradient is 10 mM/500 mM NaCl.

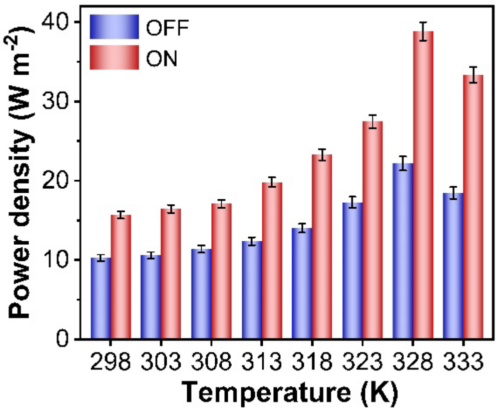

Supplementary Figure 48. Power density of PESM under different temperatures with and without light irradiation. Concentration gradient is 10 mM/500 mM NaCl. Error bars represent standard deviation of three different measurements.

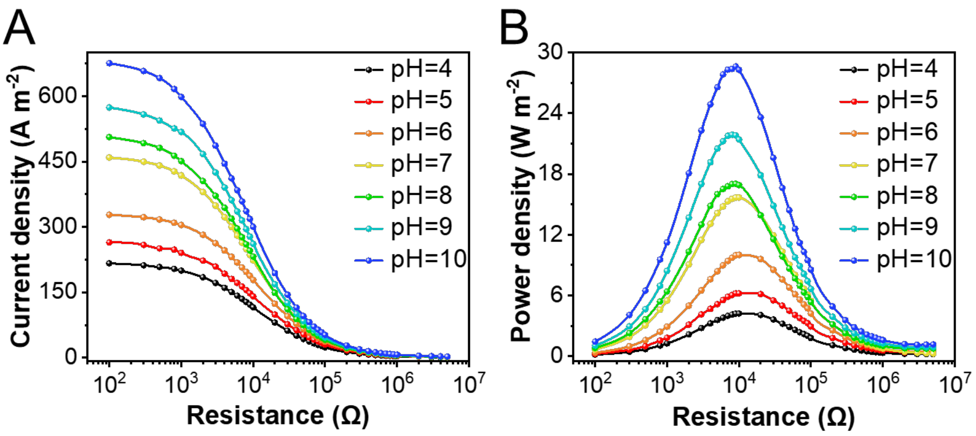

Supplementary Figure 49. (A) Current density and (B) power density of PESM with light irradiation under different pH. Concentration gradient is 10 mM/500 mM NaCl.

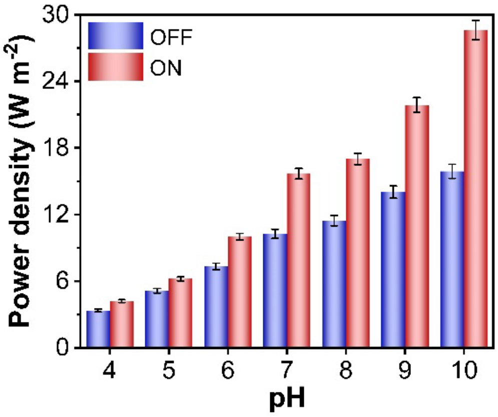

Supplementary Figure 50. Power density of PESM under different pH with and without light irradiation. Concentration gradient is 10 mM/500 mM NaCl. Error bars represent standard deviation of three different measurements.

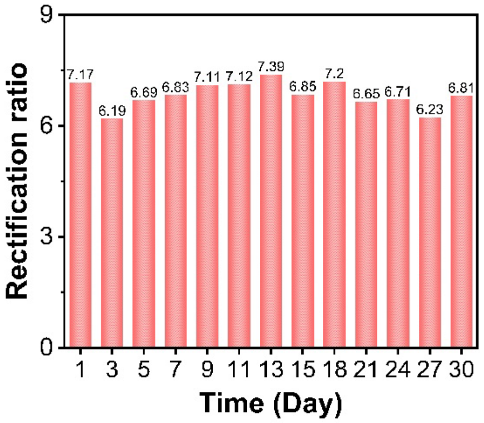

Supplementary Figure 51. Stability of PESM for ICR ratio (1 M KCl). The PESM membrane was constantly immersed in 10 mM NaCl all the time for 1-30 days, respectively.

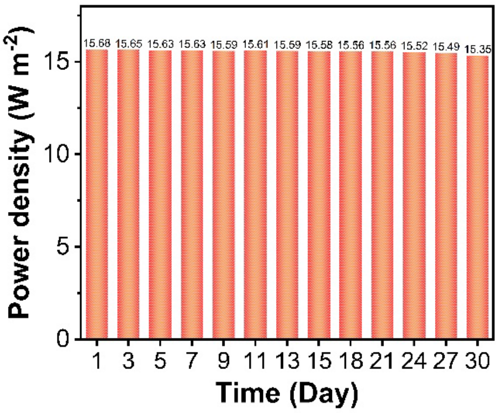

Supplementary Figure 52. Stability of PESM under irradiation for energy conversion (50-fold NaCl). The PESM membrane was constantly immersed in 10 mM NaCl all the time for 1-30 days, respectively.

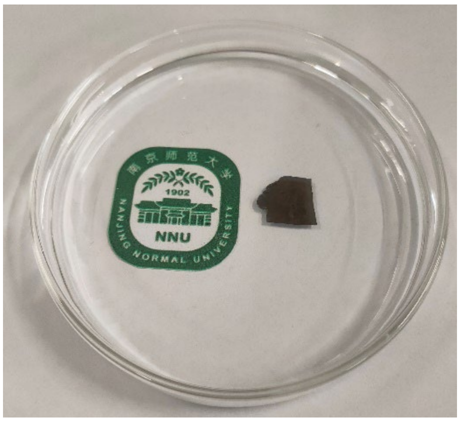

Supplementary Figure 53. The photograph of the PESM membrane after 30-day usage. The PESM membrane was constantly immersed in 10 mM NaCl all the time for 30 days.

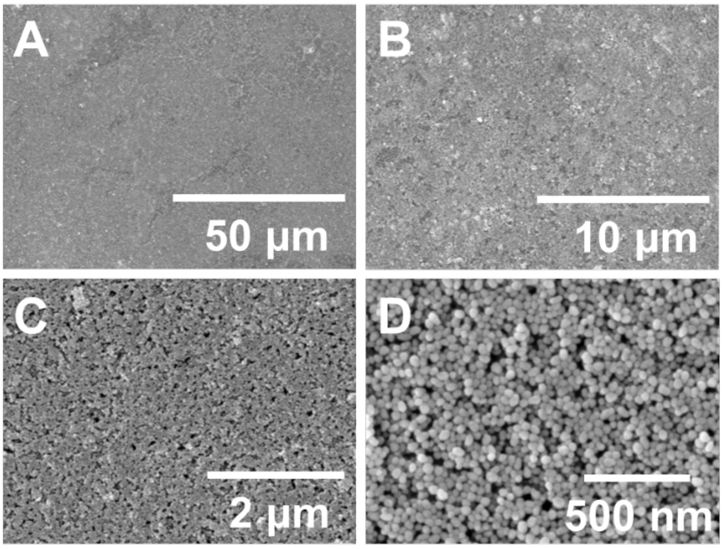

Supplementary Figure 54. (A-D) The SEM images of the PESM membrane after 30-day usage. The PESM membrane was constantly immersed in 10 mM NaCl all the time for 30 days.

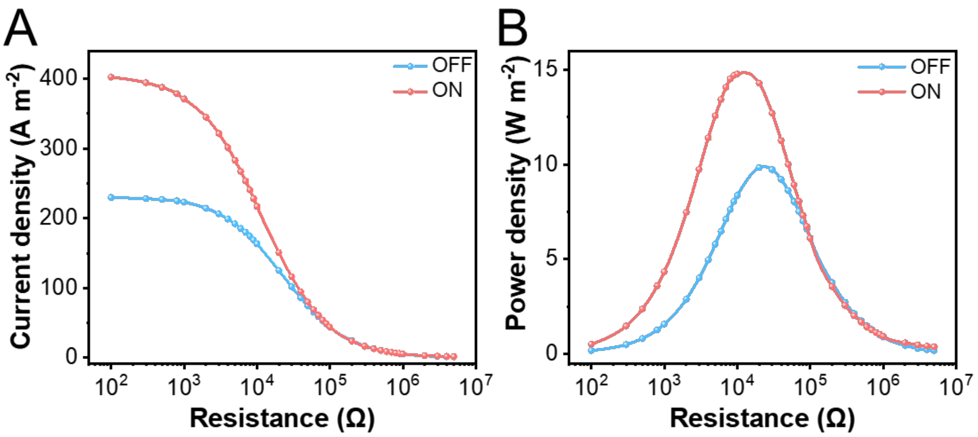

Supplementary Figure 55. (A)The current density and (B)power density of the PESM membrane under natural seawater and river water.

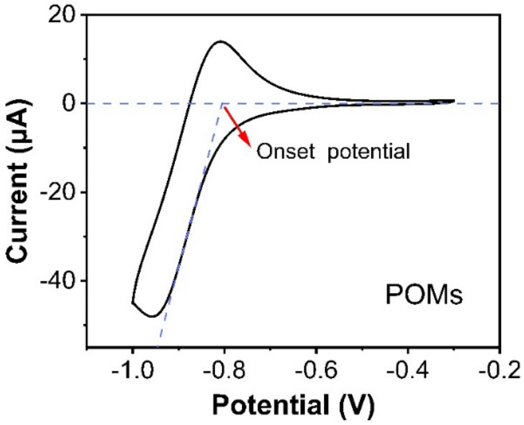

Supplementary Figure 56. Cyclic voltammogram of the POMs using an Ag/AgCl reference electrode.

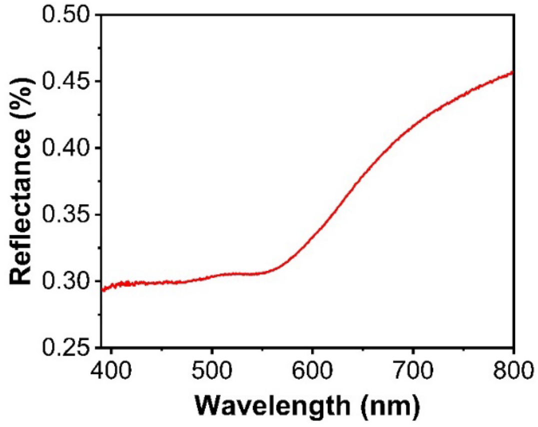

Supplementary Figure 57. UV-Vis diffuse-reflectance spectrum of POMs.

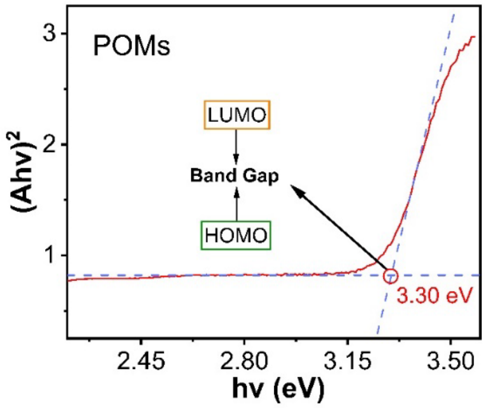

Supplementary Figure 58. Band gap of POMs estimated from UV-vis reflectance spectrum.

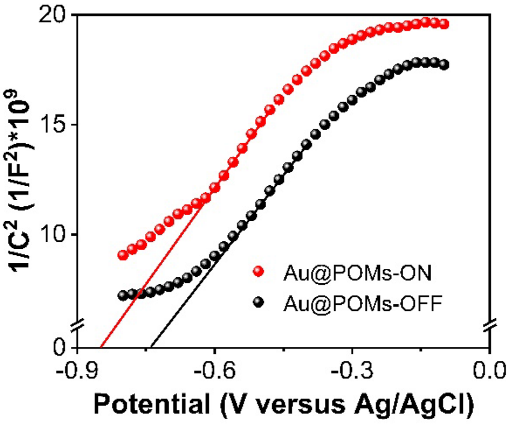

Supplementary Figure 59. Mott-Schottky plots of Au@POMs with and without light irradiation, respectively.

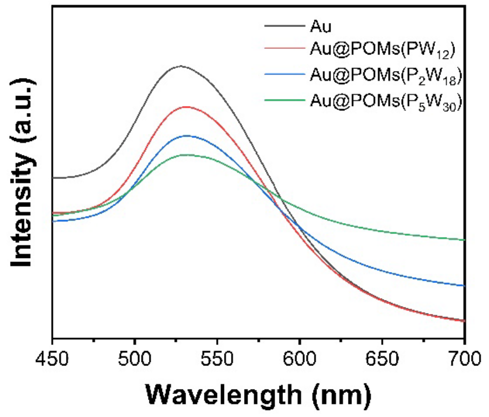

Supplementary Figure 60. UV-vis absorption spectra of AuNPs and Au@POMs (PW_{12}, P_2W_{18} and P_5W_{30}).

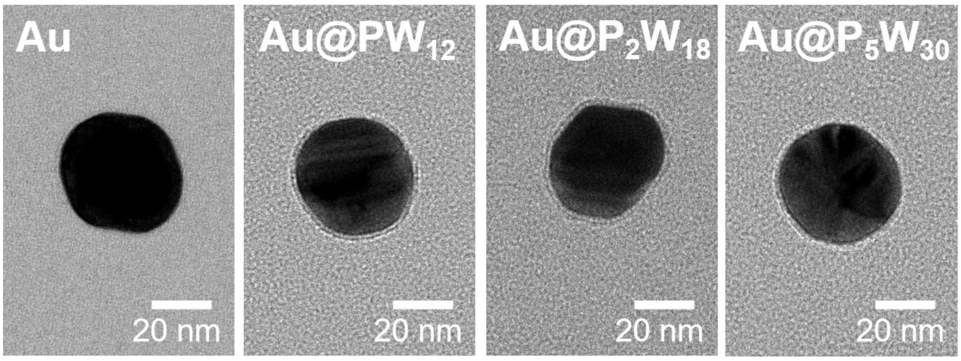

Supplementary Figure 61. TEM images of AuNPs and Au@POMs (PW_{12}, P_2W_{18} and P_5W_{30}).

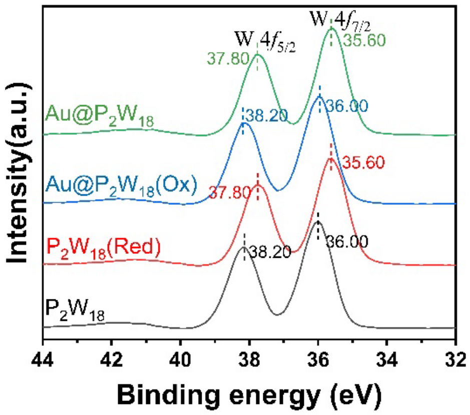

Supplementary Figure 62. The W 4f XPS spectra of P_2W_{18} and Au@P_2W_{18}.

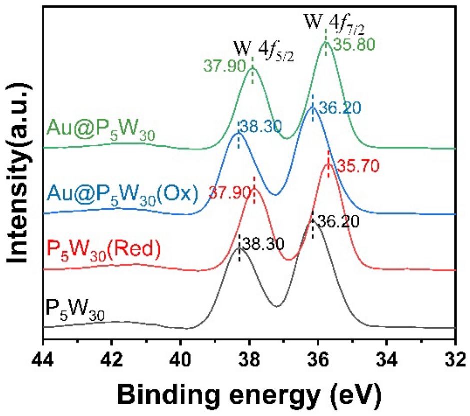

Supplementary Figure 63. The W 4f XPS spectra of P_5W_{30} and Au@P_5W_{30}.

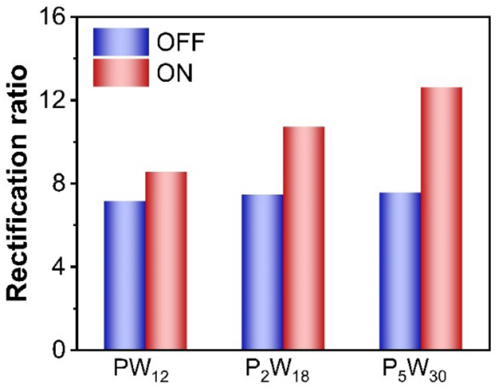

Supplementary Figure 64. ICR ratio of PESM based on Keggin-type PW_{12}, Wells-Dawson-type P_2W_{18} and Preyssler-type P_5W_{30} with and without light irradiation, respectively.

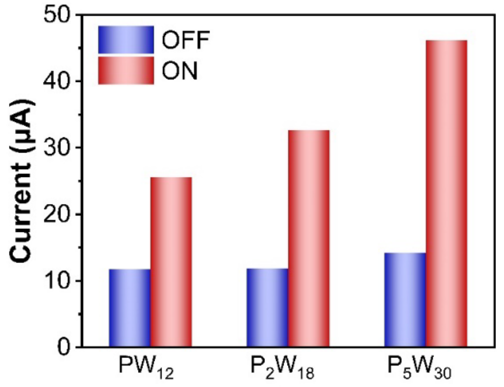

Supplementary Figure 65. Current of PESM based on Keggin-type PW_{12}, Wells-Dawson-type P_2W_{18} and Preyssler-type P_5W_{30} with and without light irradiation, respectively.

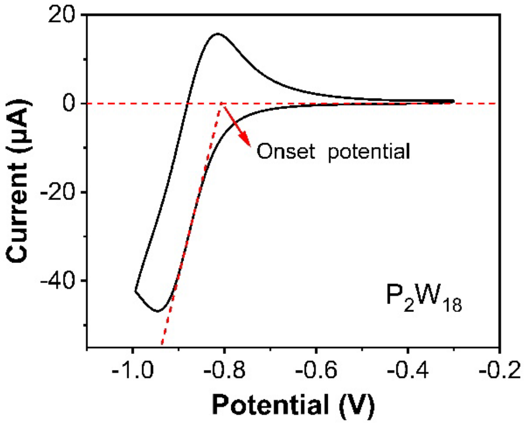

Supplementary Figure 66. The cyclic voltammogram of the POMs (P_2W_{18}) using an Ag/AgCl reference electrode.

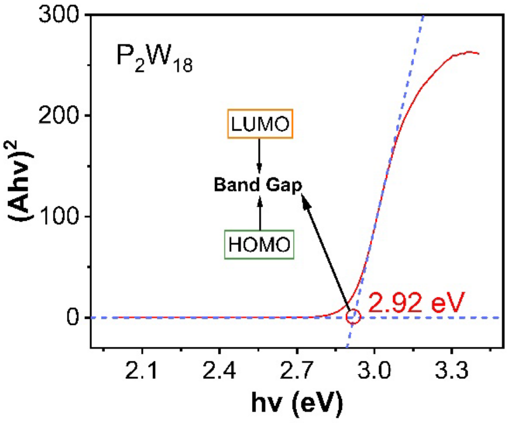

Supplementary Figure 67. The band gap of POMs (P_2W_{18}).

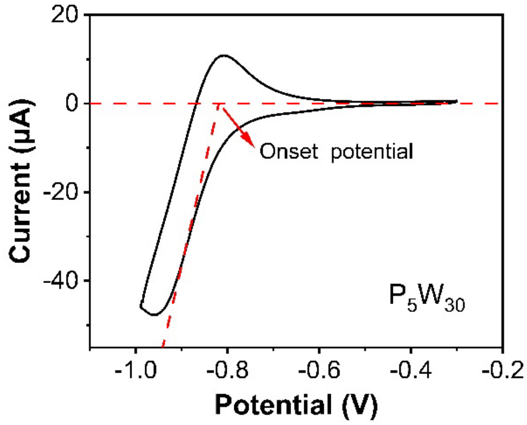

Supplementary Figure 68. The cyclic voltammogram of the POMs (P_5W_{30}) using an Ag/AgCl reference electrode.

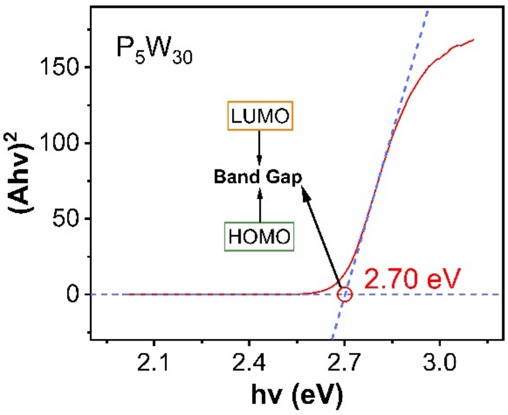

Supplementary Figure 69. The band gap of POMs (P_5W_{30}).

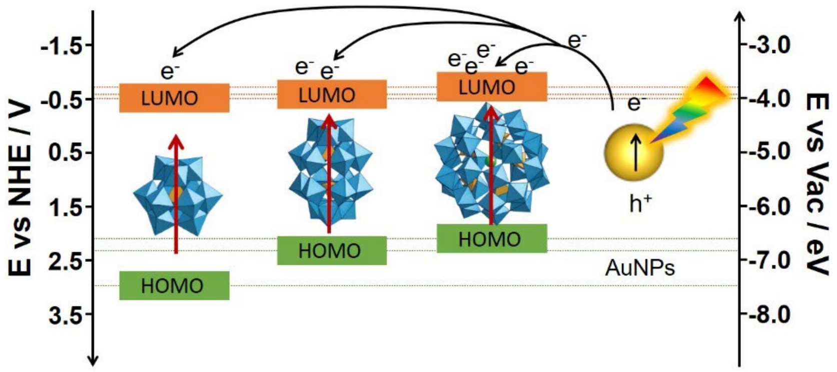

Supplementary Figure 70. Schematic and energy level diagram illuminating hot-electron injection from Au NPs to POMs (including PW_{12}, P_2W_{18} and P_5W_{30}).

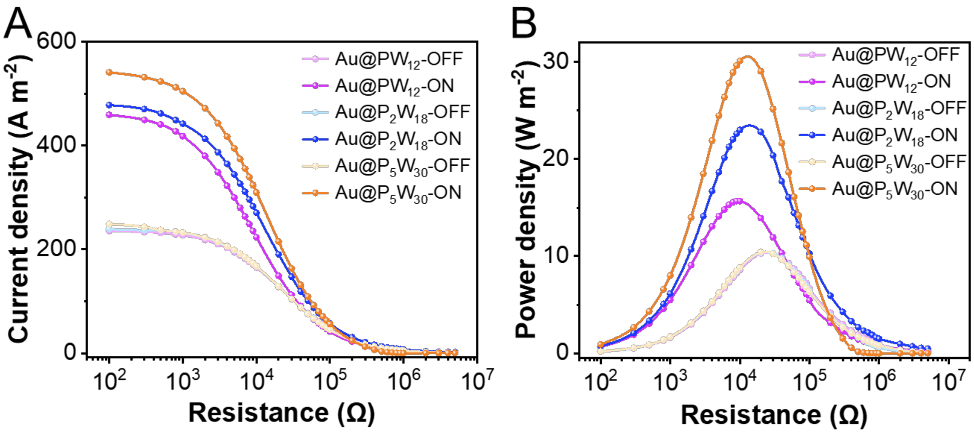

Supplementary Figure 71. (A) Current density and (B) power density of PESM based on Keggin-type PW_{12}, Wells-Dawson-type P_2W_{18} and Preyssler-type P_5W_{30} with and without light irradiation, respectively. Concentration gradient is 10 mM/500 mM NaCl.

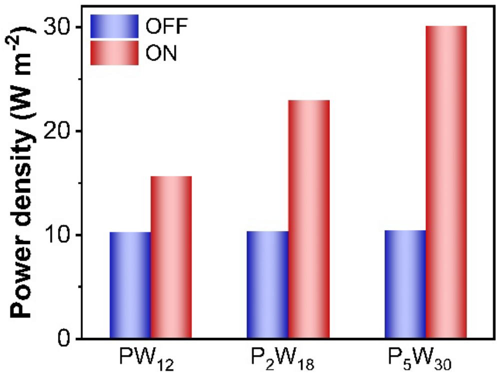

Supplementary Figure 72. Power density of PESM based on Keggin-type PW_{12}, Wells-Dawson-type P_2W_{18} and Preyssler-type P_5W_{30} with and without light irradiation, respectively. Concentration gradient is 10 mM/500 mM NaCl.

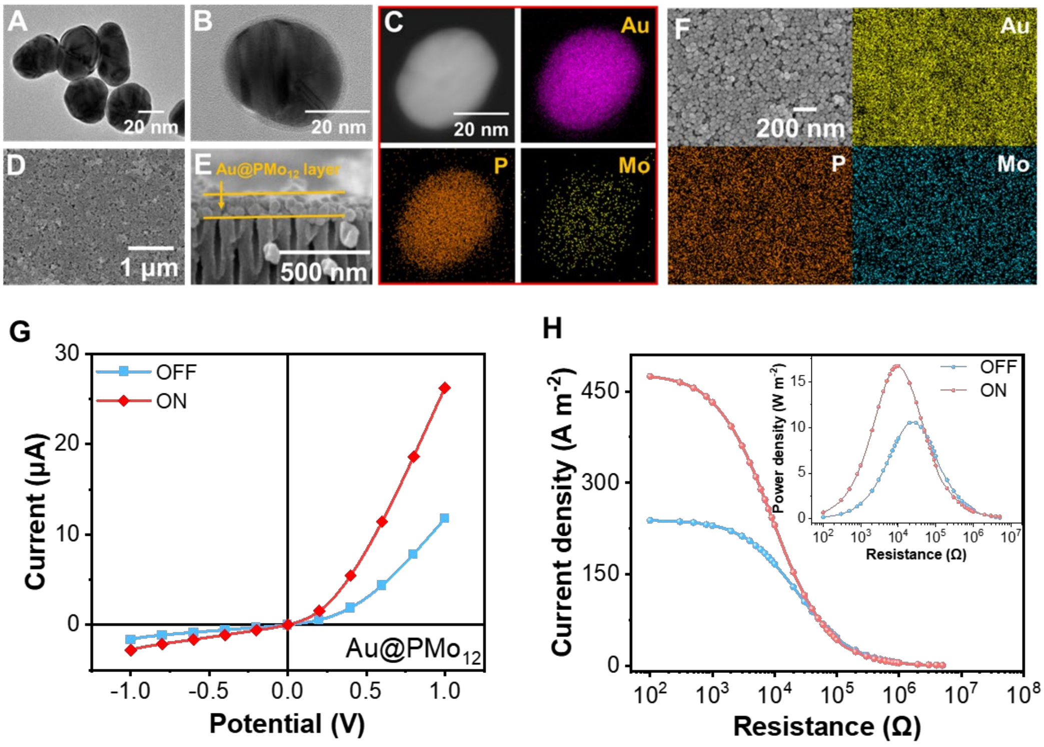

Supplementary Figure 73. (A&B) TEM images of Au@PMo_{12}. (C) The HAADF-STEM image of Au@PMo_{12} and corresponding energy-dispersive x-ray (EDX) elemental mappings of Au, P and Mo. (D) SEM image of the top of PESM based on PMo_{12}. (E) SEM image of the cross-section of PESM based on PMo_{12}. (F) SEM image and EDX elemental mappings of the top of PESM based on PMo_{12}. (G) I-V curves of PESM based on PMo_{12} in 1M KCl solutions with and without light irradiation. (H) The current and power density of PESM based on PMo_{12} with and without light irradiation (10 mM/500 mM NaCl).

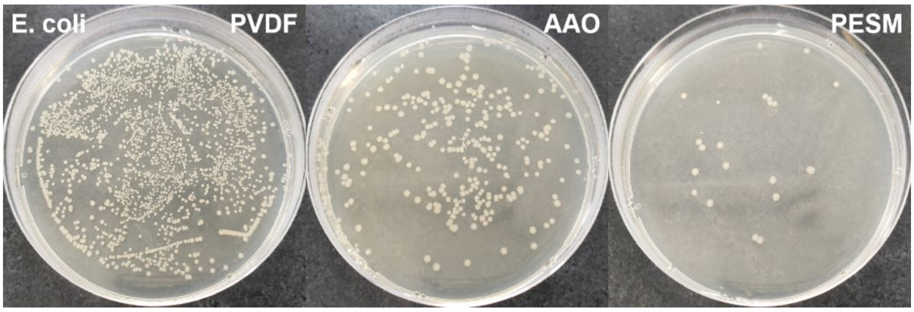

Supplementary Figure 74. Colony growth after the static bacterial adhesion test (Gram-negative bacteria-E. coli).

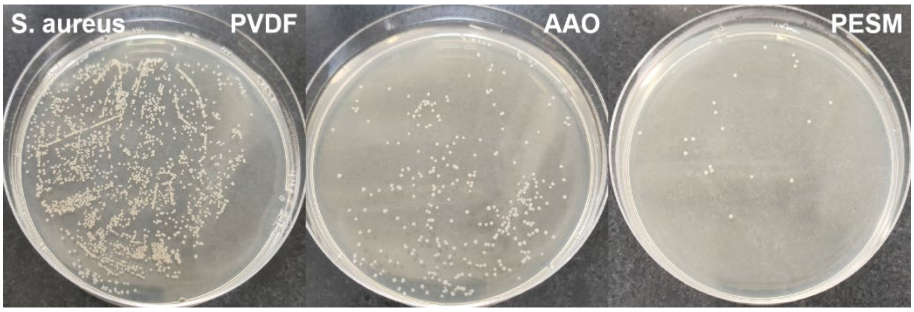

Supplementary Figure 75. Colony growth after the static bacterial adhesion test (Gram-positive bacteria-S. aureus).

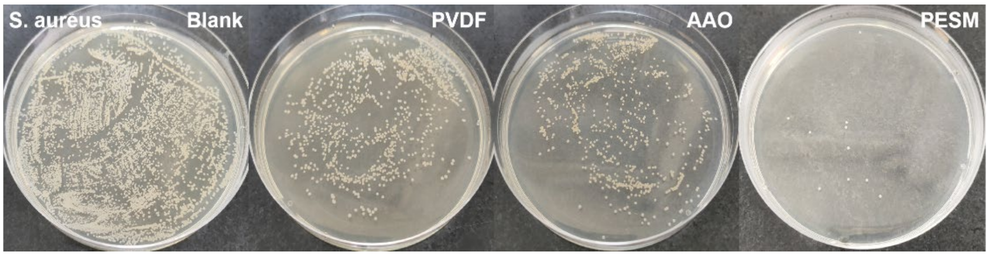

Supplementary Figure 76. Colony growth after the photoinduced germicidal test (Gram-positive bacteria-S. aureus).

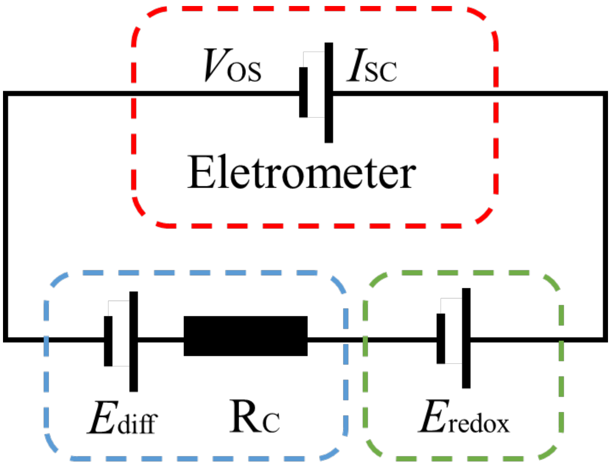

$$E_{diff} = V_{OS} + E_{redox} \qquad (1)$$

Supplementary Figure 77. Diagram demonstrated the contributions of the different parts to the overall measure I_{SC} and V_{OS}. Salinity-gradient-driven power harvesting system based on PESM. E_{redox} can be calculated as:

$$E_{redox} = \frac{RT}{zF} ln \frac{\gamma_{C_H} C_H}{\gamma_{C_l} C_l} \qquad (2)$$

R, T, z, F, and c represent separately the gas constant, temperature, charge number, Faraday constant, and mean activity coefficient.

Supplementary Table 1. Corresponding I_{SC}, V_{OC}, E_{redox}, and E_{diff} under different concentration gradients.

C_{max}/C_{min}	I_{SC}, μA	V_{OC}, mV	E_{redox}, mV	E_{diff}, mV
1(1mM/1mM)	Set to 0			
10(10mM/1mM)	1.327	91	57	34
100(100mM/1mM)	4.134	179	113	66
500(500mM/1mM)	15.54	236	150	86
1000(1000mM/1mM)	31.42	275	165	110
3000(3000mM/1mM)	48.4	315	192	123

Supplementary Table 2. Cation transfer number of PESM.

Concentration gradient(mM/mM)	10/1	100/1	500/1	1000/1	3000/1
Cation transference number	0.80	0.79	0.79	0.83	0.82

The ion transference number is calculated using the formula[1]:

$$t_n = \frac{1}{2}\left(\frac{E_{diff}}{E_{redox}} + 1\right) \qquad (3)$$

Supplementary Table 3. Comparison of output power density of PESM with previously reported nanomembranes based energy conversion systems.

Membrane type	Structure characteristics	Ion selectivity	Power density	Reference
carbonaceous ordered mesoporous nanowires/AAO (CMWs/AAO)	Heterogeneous membrane	cation selectivity	2.78 W m^{-2} C_H/C_L=50	[2]
Mesoporous carbon-silica/AAO (MCS/AAO)	Heterogeneous membrane	cation selectivity	5.04 W m^{-2} C_H/C_L=50	[3]
Graphene oxide membrane (GOM)	Homogeneous membrane	cation selectivity	5.5 W m^{-2} C_H/C_L=50	[4]
Two-dimensional molybdenum disulfifide/Cellulose nanofifiber (2D MoS_2/CNFs)	Heterogeneous membrane	cation selectivity	6.7 W m^{-2} C_H/C_L=50	[5]
Janus three-dimensional porous membrane (Janus 3D)	Heterogeneous membrane	anion selectivity	2.66 W m^{-2} C_H/C_L=50	[6]
MXene/Kevlar nanofiber composite membrane (MXene/Kevlar)	Heterogeneous membrane	cation selectivity	4.1 W m^{-2} C_H/C_L=50	[7]
Silk-based hybrid nanochannel membrane (SNF/AAO)	Heterogeneous membrane	cation selectivity	2.86 W m^{-2} C_H/C_L=50	[8]
Three-dimensional hydrogel/ aramid nanofiber (ANF/Gel)	Heterogeneous membrane	cation selectivity	506 W m^{-2} C_H/C_L=50	[9]
Porphyrin MOF membrane (p-MOF-AAO)	Heterogeneous membrane	cation selectivity	7.74 W m^{-2} C_H/C_L=50	[10]
2D sulfonated covalent organic framework/ aramid nanofiber (2D sulfonated COF/ANF)	Heterogeneous membrane	cation selectivity	9.6 W m^{-2} C_H/C_L=50	[11]
sulfonated poly (ether ether ketone)/ porous anodic alumina (SPEEK/AAO)	Heterogeneous membrane	cation selectivity	4.8 W m^{-2} C_H/C_L=50	[12]
a free-standing covalent	Homogeneous	cation	5.9 W m^{-2}	[13]

organic framework membrane (COFs)	membrane	selectivity	C_H/C_L=50	
$Ti_3C_2T_x$ MXene	Homogeneous membrane	cation selectivity	12.8 W m^{-2} C_H/C_L=50	14
Graphene oxide modified with polyamide/ Anodic aluminium oxide (PA-GO/AAO)	Heterogeneous membrane	cation selectivity	3.73 W m^{-2} C_H/C_L=50	15
covalent organic polymers membrane (COPs)	Homogeneous membrane	cation selectivity	6.21 W m^{-2} C_H/C_L=50	16
Plasmonic Electron Sponge Nanomembrane (PESM)	Heterogeneous membrane	cation selectivity	30.11 W m^{-2} C_H/C_L=50	This work

Supplementary Table 4. TEM transmission electron microscopy (TEM) combined with energy-dispersive X-ray spectroscopy (EDS) analysis of Au@POMs using PW_{12}, P_2W_{18} and P_5W_{30} as ligands, respectively[17].

	PW_{12}			P_2W_{18}			P_5W_{30}		
Mass Fraction of Au (%)	93.9	94.24	94.34	93.25	93.51	93.48	93.04	93.07	92.96
Mass Fraction of W (%)	6.1	5.76	5.66	6.75	6.49	6.52	6.96	6.93	7.04
The Number of POMs Loaded around AuNPs	7688	7234	7101	5711	5476	5503	3541	3525	3585
The Average Number of POMs	7341			5563			3550		
The Number of Electrons Recepted per Cluster	12 e^-			18 e^-			30 e^-		
The Maximum number of electrons per Au@POMs	88092 e^-			100134 e^-			106500 e^-		

The Number of POMs Loaded around AuNPs is calculated using the formula:

$$N = \frac{\rho(Au)\times V(Au)\times N_A\times \omega(W)}{M(W)\times \omega(Au)\times N(W)} \quad (4)$$

V(Au) represent the volume of one AuNPs. M(W) represent the atomic mass of W. N(W) represent the number of W atom in one POMs.

Based on the energy-dispersive X-ray spectroscopy analysis of Au@POMs (with different ligands: PW_{12}, P_2W_{18} and P_5W_{30}), the loading capacity of POMs was evaluated by the mass fraction of Au and W, and the detailed calculation process was provided in Supplementary Table 4.[17] The average number of PW_{12} per AuNP was calculated to be approximately 7341. Similarly, the average number of P_2W_{18} and P_5W_{30} per AuNP was calculated to be approximately 5563 and 3550, respectively. Correspondingly, the level of electrons storage capacity of PW_{12}, P_2W_{18} and P_5W_{30} layer were determined to be 88092, 100134, and 106500 e^-, respectively. It indicated that the potential charge density of Au@P_2W_{18} and Au@P_5W_{30} was theoretically higher than Au@PW_{12}.

References

1. Yu, C., Zhu, X., Wang, C., Zhou, Y., Jia, X., Jiang, L., Liu, X., Wallace, G. G. A smart cyto-compatible asymmetric polypyrrole membrane for salinity power generation. *Nano Energy* **53**, 475-482 (2018).

2. Xie, L., Zhou, S., Liu, J., Qiu, B., Liu, T., Liang, Q., Zheng, X., Li, B., Zeng, J., Yan, M., He, Y., Zhang, X., Zeng, H., Ma, D., Chen, P., Liang, K., Jiang, L., Wang, Y., Zhao, D., Kong, B. Sequential Superassembly of Nanofiber Arrays to Carbonaceous Ordered Mesoporous Nanowires and Their Heterostructure Membranes for Osmotic Energy Conversion. *J. Am. Chem. Soc.* **143**, 6922-6932 (2021).

3. Zhou, S., Xie, L., Li, X., Huang, Y., Zhang, L., Liang, Q., Yan, M., Zeng, J., Qiu, B., Liu, T., Tang, J., Wen, L., Jiang, L., Kong, B. Interfacial Super-Assembly of Ordered Mesoporous Carbon-Silica/AAO Hybrid Membrane with Enhanced Permselectivity for Temperature- and pH-Sensitive Smart Ion Transport. *Angew. Chem. Int. Ed.* **60**, 26167-26176 (2021).

4. Qian, Y., Liu, D., Yang, G., Wang, L., Liu, Y., Chen, C., Wang, X., Lei, W. Boosting Osmotic Energy Conversion of Graphene Oxide Membranes via Self-Exfoliation Behavior in Nano-Confinement Spaces. *J. Am. Chem. Soc.* **144**, 13764-13772 (2022).

5. Zhu, C., Liu, P., Niu, B., Liu, Y., Xin, W., Chen, W., Kong, X. Y., Zhang, Z., Jiang, L., Wen, L. Metallic Two-Dimensional MoS2 Composites as High-Performance Osmotic Energy Conversion Membranes. *J. Am. Chem. Soc.* **143**, 1932-1940 (2021).

6. Zhu, X. b., Hao, J. r., Bao, B., Zhou, Y. h., Zhang, H. b., Pang, J. h., Jiang, Z. h., Jiang, L. Unique ion rectification in hypersaline environment: A high-performance and sustainable power generator system. *Sci. Adv.* **4**, eaau1665 (2018).

7. Zhang, Z., Yang, S., Zhang, P., Zhang, J., Chen, G., Feng, X. Mechanically strong MXene/Kevlar nanofiber composite membranes as high-performance nanofluidic osmotic power generators. *Nat. Commun.* **10**, 2920 (2019).

8. Xin, W., Zhang, Z., Huang, X., Hu, Y., Zhou, T., Zhu, C., Kong, X. Y., Jiang, L., Wen, L. High-performance silk-based hybrid membranes employed for osmotic energy conversion. *Nat. Commun.* **10**, 3876 (2019).

9. Zhang, Z., He, L., Zhu, C., Qian, Y., Wen, L., Jiang, L. Improved osmotic energy conversion in heterogeneous membrane boosted by three-dimensional hydrogel interface. *Nat. Commun.* **11**, 875 (2020).

10. Li, Z. Q., Zhu, G. L., Mo, R. J., Wu, M. Y., Ding, X. L., Huang, L. Q., Wu, Z. Q., Xia, X. H. Light-Enhanced Osmotic Energy Harvester Using Photoactive Porphyrin Metal-Organic Framework Membranes. *Angew. Chem. Int. Ed.* **61**, e202202698 (2022).

11. Man, Z., Safaei, J., Zhang, Z., Wang, Y., Zhou, D., Li, P., Zhang, X., Jiang, L., Wang, G. Serosa-Mimetic Nanoarchitecture Membranes for Highly Efficient Osmotic Energy Generation. *J. Am. Chem. Soc.* **143**, 16206-16216 (2021).

12. Hou, S. h., Zhang, Q. r., Zhang, Z., Kong, X. y., Lu, B. z., Wen, L. p., Jiang, L. Charged porous asymmetric membrane for enhancing salinity gradient energy conversion. *Nano Energy* **79**, 105509 (2021).

13. Hou, S., Ji, W., Chen, J., Teng, Y., Wen, L., Jiang, L. Free-Standing Covalent Organic Framework Membrane for High-Efficiency Salinity Gradient Energy Conversion. *Angew. Chem. Int. Ed.* **60**, 9925-9930 (2021).

14. Hong, S., El-Demellawi, J. K., Lei, Y., Liu, Z., Marzooqi, F. A., Arafat, H. A., Alshareef, H. N. Porous

Ti(3)C(2)T(x) MXene Membranes for Highly Efficient Salinity Gradient Energy Harvesting. *ACS Nano* **16**, 792-800 (2022).
15. Zhang, L., Zhou, S., Xie, L., Wen, L., Tang, J., Liang, K., Kong, X., Zeng, J., Zhang, R., Liu, J., Qiu, B., Jiang, L., Kong, B. Interfacial Super-Assembly of T-Mode Janus Porous Heterochannels from Layered Graphene and Aluminum Oxide Array for Smart Oriented Ion Transportation. *Small* **17**, e2100141 (2021).
16. Zhang, C., Xiao, T., Lu, B., He, J., Wang, Y., Zhai, J. Large-Area Covalent Organic Polymers Membrane via Sol-Gel Approach for Harvesting the Salinity Gradient Energy. *Small* **18**, e2107600 (2022).
17. Xia, K., Yatabe, T., Yonesato, K., Yabe, T., Kikkawa, S., Yamazoe, S., Nakata, A., Yamaguchi, K., Suzuki, K. Supported Anionic Gold Nanoparticle Catalysts Modified Using Highly Negatively Charged Multivacant Polyoxometalates. *Angew. Chem. Int. Ed.* **61**, e202205873 (2022).
